# Development of a visual tool to assess six dimensions of health and its validation in patients with endocrine disorders

**DOI:** 10.1007/s00508-021-01809-y

**Published:** 2021-02-04

**Authors:** Christian Fazekas, Dennis Linder, Franziska Matzer, Christian Vajda, Alexander Avian, Verena Theiler-Schwetz, Christian Trummer, Julia Došen, Jelena Rokvic, Marco Mohl, Stefan Pilz

**Affiliations:** 1 Department of Medical Psychology and Psychotherapy, Medical University of Graz, Auenbruggerplatz 3, 8036 Graz, Austria; 2 Department of Internal Medicine, Division of Endocrinology and Diabetology, Medical University of Graz, Graz, Austria; 3 Institute for Medical Informatics, Statistics and Documentation, Medical University of Graz, Graz, Austria; 4 Ben Gurion University of the Negev, Beer-Sheva, Israel

**Keywords:** Biopsychosocial model, Endocrinology, Medical encounter, Psychosomatic assessment, Visual analogue scale

## Abstract

**Background:**

Psychosocial factors significantly influence patient care in many fields of medicine, among these in the field of endocrinology. Easily applicable validated assessment tools for such psychosocial factors are lacking. Visual instruments may facilitate doctor-patient communication. This study describes the development and validation of a multidimensional visual tool for the self-assessment of health.

**Methods:**

An expert panel performed the multistep development of the psychosomatic assessment health disc (PAHD). Assessment of face validity was performed by means of a focus group of medical doctors (*n* = 6) and patient interviews (*n* = 24). For determining test-retest reliability, internal consistency and construct validity, patients of an endocrine outpatient clinic in Graz, Austria, completed the PAHD and the following questionnaires: short-form 36 health survey, work ability index, Pittsburgh sleep quality index and the social life scales of the life satisfaction questionnaire.

**Results:**

A numeric six-item analogue scale was developed in the form of a disc. It addresses the following aspects of health: physical well-being, social life, sexuality, mental well-being, sleep, working ability/performance. For the validation process, 177 patients (57.1% females) participated in the study. Correlation coefficients of the six items with other questionnaires ranged between r = 0.51 (social life) and r = 0.72 (sleep). Test-retest reliability was assessed among 98 patients and was ≥ 0.74 for all 6 items, while Cronbach’s alpha was 0.78.

**Conclusion:**

The psychometric properties of the PAHD support its use in clinical encounters with patients suffering from endocrine disorders. Further validation studies may be required to extend its application to other fields of medicine.

**Supplementary Information:**

The online version of this article (10.1007/s00508-021-01809-y) contains supplementary material, which is available to authorized users.

## Introduction

Psychosocial issues may put a heavy burden on patients with endocrine disorders. Several guidelines for various endocrine diseases recommend taking psychosocial aspects of health into account for treatment decisions [1–6]; however, routine assessment of such issues appears to be largely missing outside clinical trials. There seem to be multiple barriers to the integration of psychosocial factors in medicine [7, 8]. This may also partly be due to the scarcity of easily applicable and validated tools. Existing questionnaires tackling psychosocial issues are often long, their administration is time consuming and their focus is often too disease-specific, so they may not adequately focus on psychosocial aspects of health which are relevant in endocrinology, such as daily life issues, psychological and sexual health. Consequently, in common practice, the current state of health may either not be addressed at all during the doctor-patient interview or, if addressed, it may occur in an unstructured and insufficient way.

The biopsychosocial model of health and disease [9] provides a theoretical framework on the basis of which psychosomatic medicine is practised: psychosomatic diagnostic approaches are thereby supposed to consider all dimensions of health, not only the purely biological ones. At the same time, all dimensions of health, as defined by the WHO must be taken into account [10]. Therefore, in a psychosomatic assessment procedure, shortcomings in physical, psychological and social well-being should be explored.

This approach also encompasses the impact of salutogenesis [11] and the patient’s health literacy [12] by taking the physical, psychological and social dimension of health into account. According to the biopsychosocial model, maintenance of health requires sufficient autoregulatory and conscious self-regulatory capacity in dealing with the dynamics of person-environment interaction. Therefore, in a psychosomatic interview, assessing biographical and current psychosocial factors which may affect individual vulnerability to disease and hinder self-regulatory competence is of foremost importance [13].

A visual instrument, such as the one presented in this paper, specifically and routinely administered during a clinical encounter, e.g. in this case in the setting of an endocrine outpatient clinic, may constitute a substantial improvement with respect to the situation outlined above.

In the domain of dermatology [14, 15] the use of specific visual tools has been reported to yield considerable advantages in patient care. In detail, a visual assessment tool which is applied during clinical interviews to assess dimensions of health may provide the following benefits:

1. Both doctor and patient will be stimulated to address issues otherwise neglected in an unstructured interview, the latter being anyway bound to take place under time constraints.
2. The visualization process will facilitate verbalization and conceptualization of aspects of the illness from the part of the patients, which may otherwise remain unaddressed and mistakenly judged as unrelated to the illness.
3. A visualization of various psychosomatic aspects of the patients’ lives is likely to provide a feeling of control on the disease to the patient for two reasons [16]. First, the resulting visualized pattern obtained during each interview implies a symbolization of areas sparsely impacted by the disease and, secondly, with respect to areas presumably influenced by the disease, the patients are enabled to see the effects of the treatment, represented as the changes of the pattern over time.
4. Also, for the medical doctor the effects of treatment on perceived health status will immediately become visible and thus provide a simple way of checking the perceived effectiveness of the medical treatment. Furthermore, the longitudinal outcome of medical interventions will easily be monitored.

Motivated by these potential advantages of such an assessment tool it was decided to create a visual instrument for addressing some specific dimensions of health from a biopsychosocial model perspective. Such an instrument should allow scoring by the patient during the doctor-patient interview within a minute. For the construction of the novel assessment tool, the Psodisk, a visual instrument developed in 2012, which aims at assessing the burden of disease in patients with psoriasis, provided substantial inspiration [14, 17]. In the case of the Psodisk, answers to 10 questions enable the acquisition of a visual representation of the current impact of psoriasis on the patient’s life in the form of a polygon during each encounter with the dermatologist [14].

### Study aims

The aim of the study was to (1) develop a visual assessment tool for self-reported data on a selected group of dimensions of health in patients with endocrine disorders. This tool should be applicable in other medical disciplines as well, clearly after demonstrating its validity and reliability in these further patient groups. As part of the developmental process, comprehensibility and practicability of the prototype (2) was to be tested in order to assess face validity. After completing all developmental steps the aim was to (3) analyze reliability including internal consistency and test-retest reliability and (4) probe for construct validity of the novel visual tool in a cohort of patients from an endocrine outpatient clinic. Finally, (5) the visual instrument was planned to be translated from German into English.

## Methods

**Fig. 1:**
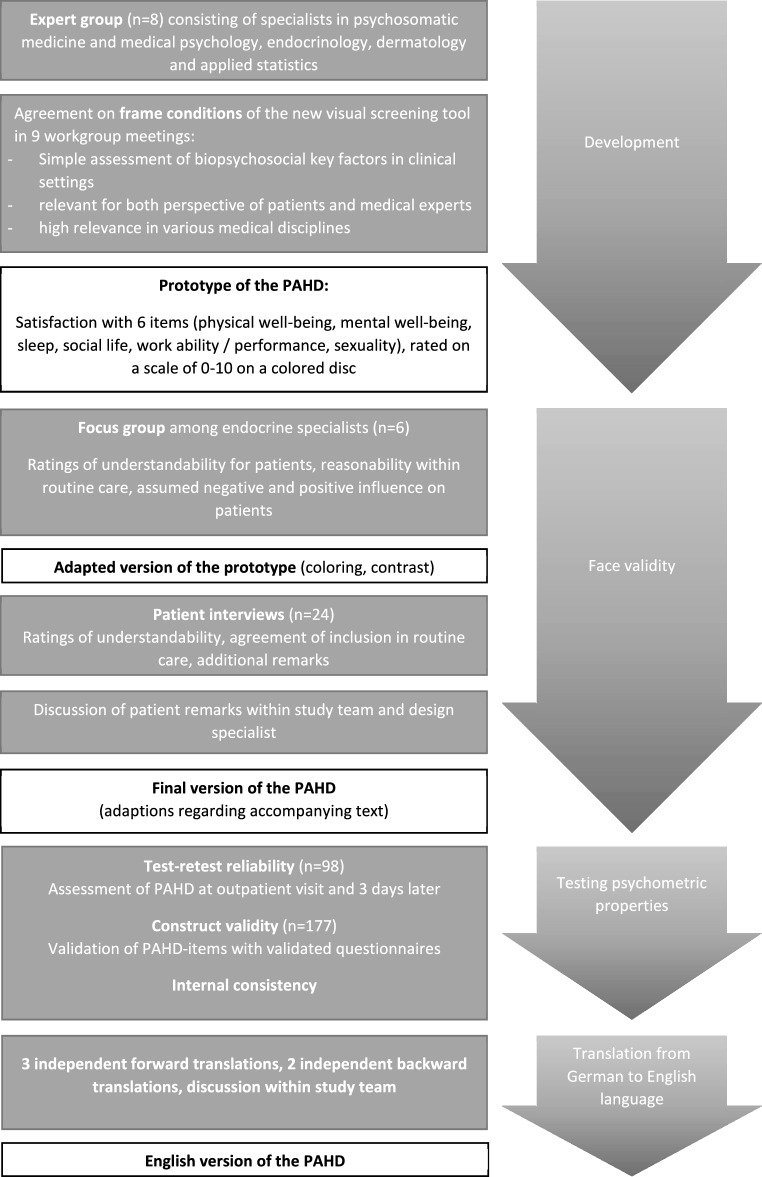
Flow chart of the study process

For the development and psychometric testing of patient-reported outcome measures (PROMs), COnsensus-based Standards for the selection of health Measurement INstruments (COSMIN) have been developed in the course of the last years [18, 19]. The COSMIN study design checklist [20] is based on the original COSMIN checklist and the COSMIN risk of bias checklist for PROMs [18, 19] and is recommended as a tool for designing studies to evaluate measurement properties of existing PROMs. In the following description of the development of the novel visual tool and its validation, the following core elements—that are also addressed by the COSMIN checklist—are specified: development of the instrument, face validity, reliability (test-retest reliability, internal consistency) and construct validity. Fig. 1 gives an overview of the study process.

### Multistep development of the instrument

The instrument was developed by an interdisciplinary expert group consisting of eight specialists in psychosomatic medicine (CF), medical psychology (FM), psychiatry (CV), endocrinology (SP, VT-S, CT), dermatology (DL) and applied statistics (AA). In a series of nine work group meetings, participants of the expert group came to the conclusion that the new visual psychosomatic screening tool should only include key factors related to all medical disciplines represented in the study group. The aim was to obtain a tool as simple as possible, which would nonetheless provide a comprehensive and useful set of biopsychosocial information. It was therefore aimed to develop an instrument that could help to routinely and comprehensively, yet, at the same time swiftly assess core health-related factors in a clinical setting. Following criteria were applied defining health-related key factors: the selected factors were to be (1) highly relevant with respect to the patient’s subjective health, health-related quality of life and well-being, and (2) highly relevant from the medical expert’s perspective. Indeed, the selected factors were to be recognized as being frequently influenced both by endocrine disorders as well as by their treatment, or to be recognized as significantly contributing to the illness experience and disease burden [12]. In addition (3), the selected factors were to show broad and frequent health-related relevance in various medical disciplines.

In accordance with this predefined approach, following factors were chosen as health-related key dimensions since they fulfilled all three criteria: physical well-being, mental well-being, sleep, social life and work ability/performance. In a next step it was decided to add sexuality as a further item: sexuality is rarely explicitly explored in clinical encounters and seems to fulfil all predefined criteria, e.g. side effects of drugs and endocrine therapies can influence sexual life satisfaction [21, 22]. After a consensus decision on this list, a consistent way to score these dimensions had to be developed. As opposed to the Psodisk, where high scores indicate a heavy impact of the disease on the patients’ lives, it was decided to present the selected health-related dimensions homogeneously as positive, i.e. higher scores were to indicate better subjective situations. This would foster the activation of health-related resources and of well-being within future regular self-assessments rather than repeatedly raising awareness of subjective deficits in clinical encounters. Thus, the tool would include a positive general question to the patient: “how satisfied are you currently with respect to the following areas of life? Please assess the degree of your satisfaction in various areas of life on a scale of 0–10, 0 being not satisfied at all and 10 being very satisfied. Please tick the number that most corresponds to your satisfaction, basing your assessment on the last 4 weeks.” In analogy to the Psodisk instrument, the answers would then be marked on a colored disc, finally allowing drawing of a hexagon. The area of the obtained hexagon would therefore increase whenever the self-reported situation of the patient would improve.

In a further step during the development of the instrument, several visual prototypes of the disc were proposed and discussed in the series of work group meetings until unanimous agreement on a final prototype version occurred. Subsequently, approval of the ethics committee was obtained to test the chosen prototype regarding its face validity, reliability and structural validity in the clinical setting. The instrument was termed psychosomatic assessment health disc (PAHD).

### Face validity

According to the COSMIN criteria, face validity can be assessed by asking patients and professionals about the relevance, comprehensiveness and comprehensibility of the items, response options, and instructions [20]. Face validity was determined by conducting an expert focus group and patient interviews. Medical comprehensibility and practicability were separately tested among medical doctors and patients of an endocrine outpatient clinic. The focus group was led by two specialists in psychosomatic medicine (CF) and medical psychology (FM) with significant background in group moderation. In the focus group, six internal medicine specialists who had not participated in the development of the prototype of the new instrument were briefly instructed about the purpose of the meeting and invited to rate the scales of the visual prototype applying it to their own persons: they answered a short, structured questionnaire on a 5-point scale ranging from 1 (strong disagreement) to 5 (strong agreement) on the following four topics: (1) the novel instrument is easy understandable for patients, (2) it is reasonable to include it in routine care, (3) implementing the instrument in clinical practice may have negative consequences for patients, (4) implementing the instrument in clinical practice may have positive consequences for patients. Finally, participants in this focus group provided additional written comments on the instrument. These answers constituted the basis for an ensuing discussion and further adaptions of the prototype. The facilitators of the focus group took notes of the topics and outcome of the group discussion.

In a further step, outpatients of the department of endocrinology were interviewed by two MDs and one psychologist (CF, CV, FM) with experience in psychosomatic medicine and endocrinology. Patients filled in the adapted version of the instrument and answered a semi-structured questionnaire containing questions 1 and 2 as described above, as well as an open question enabling additional remarks.

### Assessment of reliability and construct validity

#### Target sample

For the validation of the final version of the PAHD, outpatients with endocrine disorders of the Division of Endocrinology and Diabetology of the Department of Internal Medicine, Medical University of Graz, were selected and contacted on the basis of a stratified approach (age groups and gender) and invited to participate in the study. Recruitment in the study was dependent on the time availability of the investigators to include study participants so that our sample should be considered a convenience sample. Nonetheless, on the days of study when the investigators were available for the recruitment of patients, all patients attending the outpatient clinic were consecutively offered recruitment.

Inclusion criteria were willingness to sign the informed consent form and age of at least 18 years. While waiting for their endocrine consultation, recruited patients filled in the new instrument and a set of questionnaires.

#### Test-retest reliability

For assessment of test-retest reliability patients were further handed over a blank version of the new instrument marked by a study code for pseudonymisation, together with a pre-paid envelope, and were asked to fill it in a second time after 2–3 days (time point two) and return it by mail. It was assumed that the time interval of 2–3 days after the outpatient visit was long enough to prevent recall biases, but short enough to ensure a stable condition of patients.

Sample size considerations were based on the assumption that a test-retest reliability of r ≥ 0.8 is desirable. When observing a correlation coefficient of r = 0.85, 113 participants had to be included to get a lower bound of the 95% confidence interval (95% CI) of 0.80. Furthermore, a drop-out of 30% from time point one to time point two was assumed. Therefore, 162 participants had to be recruited to reach a sample size of 113 at the second time point.

#### Construct validity

For validity evaluations of the PAHD the following questionnaires and tests were used:

##### Short form health survey (SF-36)

The 36-item short form health survey (SF-36) is a 36-item multidimensional questionnaire assessing different aspects of health-related quality of life (HrQoL). The HrQoL is represented in eight scales (physical functioning, role physical, bodily pain, general health, vitality, social functioning, role emotional, and mental health) that can also be mapped in a physical component summary and a mental component summary score. Raw scores are transformed into scales ranging from 0–100 according to the SF-36 user’s manual. Operation time of the questionnaire is about 10 min; reliability and validity of the SF-36 are well documented [23].

##### Questionnaire on life satisfaction (FLZ)

The questionnaire on life satisfaction (*Fragebogen zur Lebenszufriedenheit, *FLZ) is a German instrument assessing different aspects of life satisfaction. From the original ten scales the following four scales were selected for the present study: friends/acquaintances, marriage and partnership, relationship to one’s children, and sexuality. Each scale consists of seven items that are rated on a 7-point scale ranging from very satisfied to very unsatisfied. For the selected scales, the processing time is about 4min. Validity of the FLZ is documented as good [24].

##### Pittsburgh sleep quality index (PSQI)

The Pittsburgh sleep quality index (PSQI) is a self-report questionnaire assessing sleep quality over the last 4 weeks that can be answered within 5min. It consists of 19 items measuring several aspects of sleep; out of these 19 items 7 component scores can be calculated (subjective sleep quality, sleep latency, sleep duration, habitual sleep efficiency, sleep disturbances, use of sleeping medication, and daytime dysfunction) as well as a global PSQI score ranging from 0 to 21, with lower scores reflecting a better sleep quality. The test-retest reliability of the total score is between 0.82 and 0.89 [25, 26], internal consistency is between 0.77 and 0.80 [27], and good validity has been documented [28].

##### Work ability index (WAI)

This test evaluates the ability to work and is used to maintain, restore and promote work ability, e.g. in occupational medicine [29]. It represents the subjectively perceived ability to work, taking into account the demands of work as well as the worker’s health status and resources. The WAI consists of ten questions, which cover physical and psychological work requirements, the state of health and the personal performance reserves; it can be answered within 5min. The items can be allocated to seven dimensions of work ability and an overall score that can reach values between 7 and 49. Work ability is reflected by the sum score and can be interpreted as poor (7–27) moderate (28–36), good (37–43) or very good (44–49). The WAI has acceptable reliability [29] and good validity [30, 31].

### Translation to English language

The PAHD was developed and validated in German. The final version of the PAHD was then translated into English. First, items were translated forward twice by two independent English native speakers and a third German native speaker who was an expert in endocrinology. Backwards translations were done by two other independent translators with German mother tongue. Differences between the original and translated versions were reviewed and discussed within the study team, who agreed on a final English version of the PAHD.

### Statistical analysis

Baseline characteristics are given with median and interquartile range (IQR) or absolute and relative frequencies. In a first step, items of the new instrument were analyzed descriptively. Therefore, the number of missing data, the median, IQR, minimum and maximum of each item was calculated. To get an impression of possible ceiling or floor effects, the number of participants using the highest and lowest response category was calculated. Since the questionnaire is designed as a combination of six independent domains regarding psychosomatic health using only one item for each concept, low to medium interitem correlations were expected. These correlations were analyzed using Spearman’s rank correlation coefficient with 95% CI. Test-retest reliability was evaluated by calculating Spearman’s rank correlation coefficient with 95% CI for the responses to each item of the first and second time point. Since overall ratings or sum scores are often desired, the internal consistency of a scale was analysed by calculating Cronbach’s alpha using all six items. As the items of the new instrument should be used as single items, we did not analyze its dimensionality.

For a first estimate of validity, the correlations of the items of the PAHD with other measures of similar concepts were calculated using Spearman’s rank correlation coefficient with 95% CI. A correlation coefficient r > 0.5 was considered relevant. Regarding construct validity, the item PAHD physical condition was assumed to be correlated with SF-36 physical functioning and SF-36 physical role functioning [23], item PAHD-social life with the FLZ subscore friends/acquaintances [24], item PAHD sexuality with the FLZ subscore sexuality [24], item PAHD mental condition with SF-36 emotional role functioning [23] and item PAHD sleep with PSQI [25]. In employed participants a correlation of PAHD ability to work/performance with WAI [29] was expected, while in unemployed participants correlations with SF-36 physical functioning, SF-36 physical role functioning and SF-36 emotional role functioning were assumed [23].

## Results

### Face validity

The focus group among medical experts included six specialists for internal medicine. Participants rated questions one, two and four (comprehensibility, practicability, possible positive effects) concordantly with strong agreement (M = 5.0, SD = 0.0) and question three (possible negative effects) with strong disagreement (M = 5.0, SD = 0.0). The ensuing focus group discussion revealed high contentment with the topics addressed by the new instrument; the position of items on the disk as well as the explanation regarding ability to work/performance were discussed and favorably assessed. Critical comments concerned aspects of the design, such as color scheme and contrast as well as difficulties in intuitively capturing the concept of the disc (in some cases the middle was intuitively thought to represent the best possible answer and uncertainty became manifest as to how the numbers should be marked). Following most of the suggestions of the expert focus group, the PAHD-prototype was adapted in terms of coloring and contrast.

After adaption of the prototype, patients were asked to complete the PAHD and report their thoughts on usability and usefulness in clinical routine. Altogether 24 outpatients (16 females) participated. The mean age was 44.4 years (SD = 15.7) with a range of 18 to 68 years. Of the participants 10 were 18–39 years old, 10 were between 40–59 years and 4 participants were 60 years or older. First, participants were asked whether the PAHD was easy to understand. Answers on this topic ranged from 3 to 5 with a mean of 4.57 (SD = 0.53). Next, participants expressed their agreement / disagreement about implementing the PAHD in routine care at the endocrine outpatient clinic. With a mean of 4.87 (SD = 0.34) and a range of 4 to 5, participants strongly agreed to this issue. Finally, remarks were collected by means of an open question fostering any further comments. A total of 13 comments addressed the format of the PAHD (size of the numbers or letters too small, too many grades in the scale, critical comments concerning the colors used, and dubiety whether the numbers needed to be marked with a cross or encircled). Five participants also commented on the content (e.g. suggestions to differentiate between family and friends or whether to include hobbies as a category) and five patients simply stressed their positive perception of the PAHD. Finally, the suggestions of the patients were discussed within the study team and with a design specialist; no further adaptions were made concerning coloring and design, but in the accompanying text it was clearly stated how answers were to be marked in the disc.

### Psychosomatic assessment health disc—final version

**Fig. 2:**
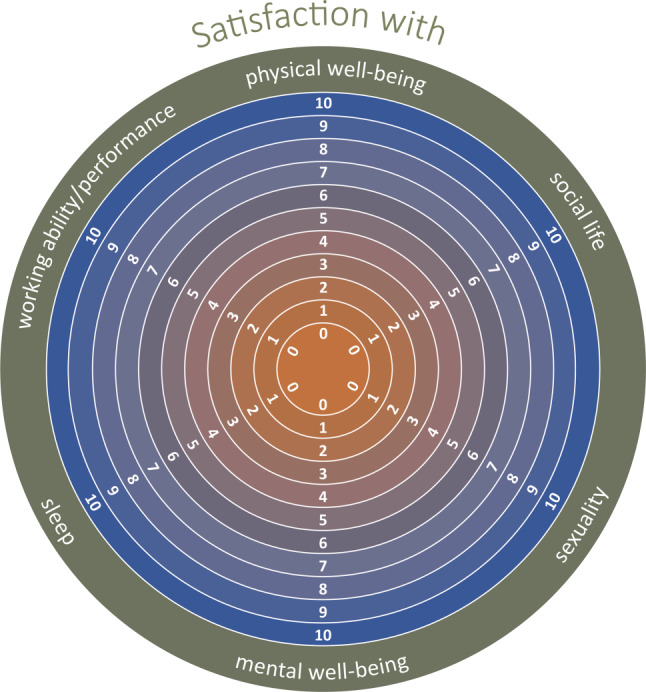
Final version of the PAHD. Translation to English was conducted based on the final German version by two English native speakers independently translating forth and back

The translated English version of the PAHD is shown in Fig. 2. The six items of the PAHD relate to physical well-being, mental well-being, sleep, social life, work ability/performance and sexuality. Participants rate their satisfaction with these domains on a scale ranging from 0 to 10, with higher numbers representing a higher degree of satisfaction. The following instruction is given: “how satisfied are you currently with the following areas of your life? Please estimate the degree of your satisfaction in different areas of life on a scale of 0–10, where 0 indicates not satisfied at all and 10 is very satisfied. Please circle the number that most closely corresponds to your satisfaction level and refer to your assessment of the last 4 weeks. Note: Please base the area of productivity on your employment. If you are not currently employed, please base productivity on your domestic activities”.

**Fig. 3:**
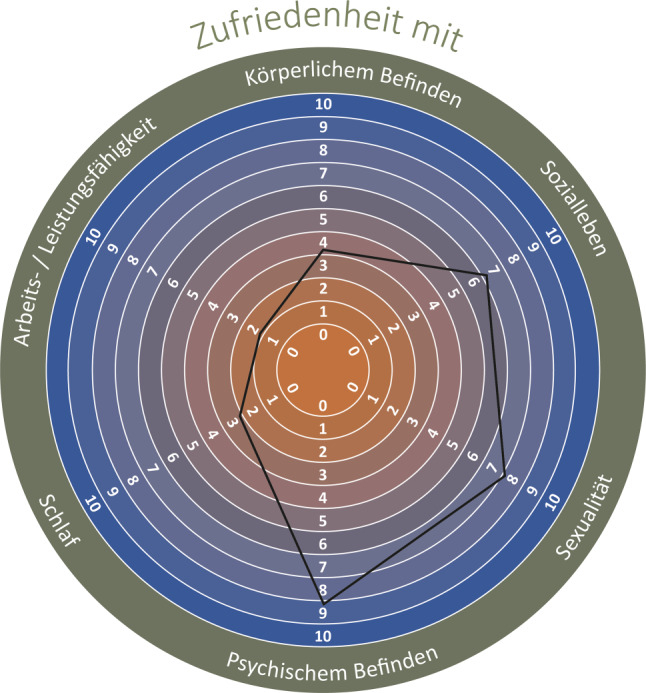
Original final version of the PAHD in German language with an example of a hexagon derived from answers to the six items

The PAHD is administered as a paper questionnaire. The original final version of the PAHD in German language is shown in Fig. 3 and includes an example of a resulting hexagon derived from answers to the six items.

### Reliability and structural validity

#### Study participants

Overall, 177 patients (median age 54 years, range 17–83 years; female 57.1%) answered the questionnaire at time point one (Table 1) and were included into the study from June 2019 to February 2020. For time point two 98 (55.4% response rate) questionnaires were returned.

**Table 1:** Sociodemographic characteristics of study participants (*n* = 177)

	Median (IQR) *n* (%)
*Age (in years)*	54 (32.5–64)
*Sex*	
Male	76 (42.9%)
Female	101 (57.1%)
*Body mass index (in kg/m*^*2*^*)*	25.5 (22.5–29.1)
*Living situation*	
Living alone	42 (23.7%)
Living with child(ren)	9 (5.1%)
Living with partner/family	115 (65.0%)
Other	6 (3.4%)
Missing	5 (2.8%)
*Marital status*	
Unmarried/alone	44 (24.9%)
Married/with partner	99 (55.9%)
Divorced	20 (11.3%)
Widowed	9 (5.1%)
Missing	5 (2.8%)
*Highest education*	
No graduation	1 (0.6%)
Primary education	22 (12.4%)
Secondary education	75 (42.4%)
Higher secondary (qualification for university entrance)	37 (20.9%)
University	37 (20.9%)
Missing	5 (2.8%)
*Occupational status*	
Full time	52 (29.4%)
Part time	30 (16.9%)
Minor	8 (4.5%)
No	69 (39.0%)
Other	12 (6.8%)
Missing	6 (3.4%)
*Household income per month*	
< 1000 €	25 (14.1%)
1000 €–< 1500 €	28 (15.8%)
1500 €–< 2000 €	26 (14.7%)
2000 €–< 2500 €	23 (13.0%)
2500 €–< 3000 €	23 (13.0%)
3000 €–< 3500 €	20 (11.3%)
3500 €–< 4000 €	10 (5.6%)
≥ 4000 €	16 (9.0%)
Missing	6 (3.4%)

All participants were in a stable clinical condition. Main reasons for attending the endocrine outpatient clinic were suspected or prevalent thyroid diseases (27% of all study participants), osteoporosis or related bone diseases (25%), while the remaining indications were largely a variety of pituitary and adrenal diseases, as well as transgender individuals. The study population represents a random sample of patients attending the endocrine outpatient clinic.

#### Descriptive statistics of PAHD data

Out of 177 respondents 5 (2.8%) did not complete all 6 items of the health disc at time point one. While for all items the response distribution tends to be skewed toward better health, all 11 response categories were used in all items except in item PAHD social life. The lowest response category was used in 0.0–6.8% of the responders and the highest in 4.0–23.7% (Table 2). At time point two 8 out of 98 (8.2%) participants did not answer all 6 items.

**Table 2:** Descriptive analysis of health disc items

	*n*	Missing	Median (IQR)	Min–max	% Highest category	% Lowest category
PAHD-1: physical well-being	177	0 (0.0%)	7 (5–8)	0–10	4.0	1.1
PAHD-2: social life	174	3 (1.7%)	8 (7–9)	3–10	23.7	0.0
PAHD-3: sexuality	173	4 (2.3%)	7 (5–9)	0–10	13.6	6.8
PAHD-4: mental well-being	175	2 (1.1%)	8 (6–9)	0–10	16.9	1.1
PAHD-5: sleep	175	2 (1.1%)	7 (5–9)	0–10	18.1	1.1
PAHD-6: working ability/performance	175	2 (1.1%)	7 (5–8)	0–10	6.8	1.7

*PAHD* psychosomatic assessment health disc

#### Test-retest reliability

Test-retest reliability was good with highest values for PAHD sleep (r = 0.86, 95% CI 0.77–0.91) and lowest for PAHD social life (r = 0.74, 95% CI 0.59–0.85) and PAHD working ability/performance (r = 0.74, 95% CI 0.62–0.84). Items showed a low to high intercorrelation with higher correlations at time point two. Highest correlations were observed between PAHD physical condition and PAHD working ability/performance (time point 1: r = 0.550, time point 2 r = 0.827) (Table 3).

**Table 3:** Inter-item correlation and test-retest reliability. Values above the diagonal are inter-item correlations of time point one. Values below the diagonal are inter-item correlations of time point two. Diagonal values are test-retest reliability values

	PAHD‑1	PAHD‑2	PAHD‑3	PAHD‑4	PAHD‑5	PAHD‑6
PAHD-1: physical well-being	*0.802*	0.240	0.228	0.425	0.351	0.550
PAHD-2: social life	0.604	*0.739*	0.416	0.530	0.287	0.353
PAHD-3: sexuality	0.457	0.496	*0.826*	0.407	0.241	0.288
PAHD-4: mental well-being	0.634	0.690	0.569	*0.769*	0.463	0.515
PAHD-5: sleep	0.657	0.349	0.326	0.489	*0.859*	0.510
PAHD-6: working ability/performance	0.827	0.546	0.332	0.632	0.699	*0.739*

*PAHD* psychosomatic assessment health disc

#### Internal consistency

Analysing all items of the PAHD together resulted in an internal consistency of Cronbach’s α = 0.78.

#### Construct validity

Associations with questionnaires evaluating similar concepts were generally good to high with the highest correlation between PAHD sleep and PSQI sleep (r = 0.72, 95% CI 0.63–0.80) (Table 4). With respect to PAHD social life, the subscale friends/acquaintances of the questionnaire on life-satisfaction provided a higher correlation with PAHD social life (r = 0.51) than the other two subscales (marriage and partnership r = 0.22, relationship to one’s children r = 0.32). The subscale friends/acquaintances was completed by 91.5% of respondents, whereas the other two subscales were answered less frequently (marriage and partnership completed by 68.4%, relationship to one’s children completed by 54.2% of respondents). Therefore, the reported correlation of the PAHD social life refers to the subscale friends/acquaintances (Table 4). Correlation with questionnaires measuring other concepts were low except for PAHD working ability/performance with PSQI (r = −0.50, 95% CI 0.37–0.62), SF-36 physical functioning (r = 0.57, 95% CI 0.46–0.67), and SF-36 physical role functioning (r = 0.54, 95% CI 0.41–0.65) and PAHD-physical well-being and WAI sum score (r = 0.53, 95% CI 0.36–0.69) (Table 4).

**Table 4:** Correlation of PAHD items with validity measures.

	PAHD‑1	PAHD‑2	PAHD‑3	PAHD‑4	PAHD‑5	PAHD‑6
SF-36 physical functioning	*0.509*	0.178	0.232	0.280	0.238	0.566
SF-36 physical role functioning	*0.460*	0.221	0.196	0.291	0.309	0.537
FLZ friends/acquaintances	0.148	*0.510*	0.313	0.380	0.201	0.214
FLZ sexuality	0.171	0.383	*0.655*	0.356	0.105	0.295
SF-36 emotional role functioning	0.270	0.343	0.307	*0.539*	0.338	0.481
PSQI	−0.399	−0.290	−0.316	−0.443	*−0.720*	−0.504
**Unemployed participant (*n*** **=** **87)**						
SF-36 physical functioning	*0.591*	0.113	0.178	0.297	0.279	*0.594*
SF-36 physical role functioning	*0.417*	0.247	0.258	0.291	0.314	*0.587*
SF-36 emotional role functioning	0.126	0.339	0.291	*0.504*	0.270	*0.441*
**Employed participants (*n*** **=** **90)**						
WAI sum score	0.531	0.486	0.268	0.451	0.35	*0.544*

*PAHD* psychosomatic assessment health disc, *SF-36* short form health survey, *FLZ* questionnaire on life satisfaction, *PSQI* Pittsburgh sleep quality index, *WAI* work ability index

## Discussion

This study reports on the development and validation of a new visual instrument for the assessment of six dimensions of health in a cohort of patients with endocrine disorders. The tool was developed with the aim of bridging the well-known gap between the theoretical implications of the biopsychosocial model and of the WHO definition of health and its transfer into daily medical practice across various medical disciplines [10, 32–35]. The psychometric properties with respect to construct validity and test-retest reliability of all six single items are good, as measured in this first validation study among outpatients with endocrine disorders. These results support the use of the novel six-item visual analogue scale for screening purposes during medical encounters in this patient group. This is further supported by the results of patient interviews and ratings that have been conducted to assess face validity. Outpatients with endocrine disorders assessed the PAHD as easy understandable and were highly in favor of having the PAHD included in routine care. These findings support the assumption that the chosen dimensions are perceived as highly relevant to patients’ subjective health and wellbeing. They further demonstrate patients’ readiness to address these aspects within the medical encounter and point at their health-related relevance within endocrinology.

A rather high Cronbach’s α of 0.78 seems to imply a link between all measured dimensions, as expected from a biopsychosocial perspective. Nevertheless, this instrument should be regarded as a multidimensional screening tool providing valid information on the following separate aspects of subjective health and well-being: physical well-being, mental well-being, social life, sleep, sexuality and working ability/performance. In addition, the hexagon resulting from the graphic representation of all six dimensions as rays of a circle, can be interpreted as a visualization of the overall state of well-being of patients at the time of the interview.

According to the recommendations of the COSMIN standards [18–20], further testing of reliability and validity of the PAHD seems desirable as an ongoing process. Among evaluations of construct validity in other patient groups, hypotheses testing could complement the validity estimations of construct validity. Furthermore, responsiveness as an indicator of longitudinal validity should be assessed for the PAHD in future studies.

The dimensions captured by the PAHD are known to be influenced by endocrine diseases and treatment [6, 36] and should therefore, as recommended by several guidelines [1–5], be regularly included in making clinical decision. On the basis of the criteria according to which the instrument was developed and on the fact that the instrument aims to measure positive, health-enhancing entities, the instrument was termed psychosomatic assessment health disc (PAHD). The name chosen points to the two main functions of this instrument: first, it should help exploring a possible link between biological and psychosocial data, and secondly, for the patient, it should help exploring satisfaction with a range of potentially disease-related dimensions. This may contribute to health literacy and self-control [16].

In more detail, an application of the PAHD during the medical encounter may have several advantages. For the medical specialist, it will facilitate collection of relevant biopsychosocial data in a standardized way, even when time resources are limited. For the patient, it will promote awareness and self-reflection concerning the multiple aspects influencing subjective health. Issues thus arising during the doctor-patient encounter, for example about specific areas of life that are perceived to be difficult, can more easily be tackled in detail in the course of the doctor-patient conversation. This may in turn result in more individualized treatment goals within patient-centered care and a corresponding increase in contextualized treatment plans, thus leading to better medical outcomes, partly by avoiding contextual errors [37–39]. Using the PAHD during follow-up consultations will allow to visually capture improvement or worsening in the biopsychosocial areas assessed. That way, it constitutes an easily applicable tool to accompany the course of treatment and evaluate treatment effects. In endocrinology, such an approach might support treatment choices in various conditions in the future. For example, in patients with thyroid function disorders, or adrenal as well as pituitary diseases, the PAHD could turn out to be useful as a complementing tool to determine and adjust the dosage of hormone-replacement therapies with respect to biopsychosocial influences. Although various disease-specific questionnaires have been developed, compared to the PAHD, they are often very time consuming, focused on a single disease entity, and sparsely capture all dimensions of the PAHD. Nonetheless, we deem that future studies of the PAHD will be needed to evaluate its congruence or lacking congruence with existing disease-specific questionnaires for the assessment of biological and psychosocial factors [32].

Finally, increased attention to psychosocial aspects in clinical routine may also contribute to the quality of the doctor-patient relationship and related outcomes such as patient satisfaction, job satisfaction of medical doctors, and patient adherence to treatment [40, 41].

It may hence be concluded that addressing psychosocial aspects of illness during the doctor-patient interview can be of crucial importance for a satisfactory outcome of the patients’ journey and positively contribute to the medical outcome.

### Limitations

Following limitations need to be considered with respect to this study and the novel instrument.

1. The development of this tool was conducted based on medical expertise in psychosomatic medicine, medical psychology, psychiatry, endocrinology, and dermatology but not in other medical fields. In addition, the validation was conducted with patients from an outpatient clinic for endocrinology. It should therefore be kept in mind that the reported validation may not apply to other patient groups; however, as the PAHD can be considered as a tool that can help put the WHO definition of health into practice, its implementation in different settings should be encouraged, particularly if accompanied by further validation studies which could support its broader use.
2. A possible gender bias should be mentioned which could have influenced the results with respect to feedback on face validity during the development of the instrument and concerning the validation study as well, as in both parts of this study more females than males were participating; however, only small differences in the results could be observed, when analyses were performed for females and males separately (supplementary tables 1 and 2). Nonetheless, further studies targeting gender differences should be conducted.
3. Psychosomatic assessment by means of the PAHD should be considered as a screening method for health-related deficits which may or may not be associated with a specific mental or physical disease. Therefore, further exploration of screening results suggesting health-related deficits seems to be required within the doctor-patient encounter to substantiate their disease-related relevance.
4. In the development of this tool it has been assumed that this health disc can be repeatedly used in a series of patient encounters without irritating or annoying the patient. It could provide longitudinal visualized data on health-related treatment effects and thus contribute to better medical outcomes. Further studies could therefore focus on how both patients and professionals perceive the inclusion of the PAHD in routine care. Besides, a randomized controlled study design will be needed to explore the impact of the repeated application of this tool on medical outcome as compared to treatment as usual. Hence, although a benefit for the medical outcome from the use of the PAHD may seem reasonable to assume, it should be kept in mind that clinical evidence for such a benefit should be provided by means of an appropriate trial.
5. The present paper does not address a very practical, yet not negligible aspect of the PAHD (and other similar instruments): psychological visual instruments are increasingly expected, for obvious reasons, to be available also in an electronic format. For the abovementioned Psodisk, and for another instrument with similar properties as the PSO-disk, the HIDRADISK [14, 15], an app was made available in Italy. Another visual instrument, called PRISM (Pictorial Representation of Illness and Self Measure) [39] is also available in a version for tablets and smartphones. Analogously, an “e-PAHD” is not yet available, but the making of an electronic version of this tool should be accomplished by this study group in the near future. An electronic version of the PAHD will not only facilitate its use in research, but also enable a visualization of changes in subjective health in the course of individual treatments.

## Conclusion

This paper reports on the successful development of a new visual self-assessment tool on the physical, psychological and social dimensions of health and its validation for a selected population of patients. As concerns validation, the PAHD showed good reliability and validity in a sample of patients with endocrine disorders. Further validation of the instrument in other clinical samples is required to confirm its broad applicability in various medical fields. The integration of the PAHD in the medical encounter could produce significant advantages for patients, medical specialists and health-related outcome. Clearly, appropriate clinical studies will be needed to support this assumed benefit.

## Supplementary Information

Supplementary Table 1: Reliability measures of PAHD in female and male study participants

Supplementary Table 2: Correlation of PAHD items with validity measures in female and male study participants
