# Supplementary material for: Development of a visual tool to assess six dimensions of health and its validation in patients with endocrine disorders
Source: Wien Klin Wochenschr. 2021 Feb 4;134(15-16):569–80. doi: 10.1007/s00508-021-01809-y (PMC9418290; doi:10.1007/s00508-021-01809-y)
Supplement: Supplementary file 1 — Supplementary Table 1: Reliability measures of PAHD in female and male study participants [file 508_2021_1809_MOESM1_ESM.docx]

Supplementary Table 1: Reliability measures

|  |  |  |
| --- | --- | --- |
| Test Retest reliability | female | male |
| PAHD-1: physical well-being | .782 (.633 - .887) | .756 (.505 - .910) |
| PAHD-2: social life | .799 (.654 - .902) | .627 (.282 - .853) |
| PAHD-3: sexuality | .831 (.679 .918) | .811 (.620 - .919) |
| PAHD-4: mental well-being | .751 (.595 - .858) | .818 (.634 - .927) |
| PAHD-5: sleep | .846 (.731 - .919) | .890 (.717 - .963) |
| PAHD-6: working ability/performance | .796 (.677 - .879) | .611 (.284 - .838) |
| Cronbach’s alpha | .770 | .785 |
