# Supplementary material for: Development of a visual tool to assess six dimensions of health and its validation in patients with endocrine disorders
Source: Wien Klin Wochenschr. 2021 Feb 4;134(15-16):569–80. doi: 10.1007/s00508-021-01809-y (PMC9418290; doi:10.1007/s00508-021-01809-y)
Supplement: Supplementary file 2 — Supplementary Table 2: Correlation of PAHD items with validity measures in female and male study participants [file 508_2021_1809_MOESM2_ESM.docx]

Supplementary Table 2a: Correlation of PAHD items with validity measures. Grey cells are correlations with questionnaires/scales measuring similar concepts. (female participants)

|  | PAHD-1 | PAHD-2 | PAHD-3 | PAHD-4 | PAHD-5 | PAHD-6 |
| --- | --- | --- | --- | --- | --- | --- |
| SF-36 physical functioning | .512^**^ | .075 | .086 | .093 | .252^*^ | .438^**^ |
| SF-36 physical role functioning | .202 | .443^**^ | .228^*^ | .599^**^ | .260^*^ | .482^**^ |
| FLZ friends / acquaintances | .236^*^ | .491^**^ | .411^**^ | .407^**^ | .200 | .362^**^ |
| FLZ_sexuality | .160 | .383^**^ | .680^**^ | .328^**^ | -.006 | .295^**^ |
| SF-36 emotional role functioning | .156 | .304^**^ | .261^*^ | .531^**^ | .223^*^ | .497^**^ |
| PSQI | -.290^**^ | -.249^*^ | -.168 | -.350^**^ | -.732^**^ | -.465^**^ |
| unemployed participant (n = 56) |  |  |  |  |  |  |
| SF-36 physical functioning | .538 | .143 | .224 | .312 | .274 | .531 |
| SF-36 physical role functioning | .413 | .237 | .274 | .328 | .263 | .611 |
| SF-36 emotional role functioning | .047 | .260 | .256 | .518 | .113 | .503 |
| employed participants (n = 45) |  |  |  |  |  |  |
| WAI sum score | .559 | .471 | .163 | .333 | .314 | .676 |

Supplementary Table 2b: Correlation of PAHD items with validity measures. Grey cells are correlations with questionnaires/scales measuring similar concepts. (male participants)

|  | PAHD-1 | PAHD-2 | PAHD-3 | PAHD-4 | PAHD-5 | PAHD-6 |
| --- | --- | --- | --- | --- | --- | --- |
| SF-36 physical functioning | .581 | .107 | .189 | .226 | .212 | .595 |
| SF-36 physical role functioning | .476 | .521 | .366 | .529 | .491 | .410 |
| FLZ friends / acquaintances | .003 | .537 | .147 | .349 | .173 | -.015 |
| FLZ_sexuality | .174 | .418 | .610 | .351 | .194 | .248 |
| SF-36 emotional role functioning | .416 | .422 | .369 | .496 | .480 | .443 |
| PSQI | -.476 | -.325 | -.491 | -.437 | -.691 | -.507 |
| unemployed participant (n = 31) |  |  |  |  |  |  |
| SF-36 physical functioning | .673 | .098 | .121 | .240 | .282 | .670 |
| SF-36 physical role functioning | .406 | .297 | .241 | .202 | .411 | .542 |
| SF-36 emotional role functioning | .282 | .471 | .334 | .464 | .567 | .411 |
| employed participants (n = 45) |  |  |  |  |  |  |
| WAI sum score | .483 | .542 | .396 | .563 | .361 | .424 |
